# Specific Features of Juvenile Idiopathic Arthritis Patients’ Cytokine Profile

**DOI:** 10.3390/biomedicines12010135

**Published:** 2024-01-09

**Authors:** Daria I. Kozlova, Arseny V. Rybakov, Karina A. Yureva, Vitaly V. Khizha, Lybov S. Sorokina, Mikhail M. Kostik, Alexandr B. Guslev

**Affiliations:** 1 Saint-Petersburg Clinical Hospital of the Russian Academy of Sciences, Saint-Petersburg 194017, Russia; aguslev@mail.ru; 2 Sechenov Institute of Evolutionary Physiology and Biochemistry of the Russian Academy of Sciences (IEPhB RAS), Saint-Petersburg 194223, Russia; aribakoff@gmail.com (A.V.R.); khizhaspb@gmail.com (V.V.K.); 3 Institute of Biomedical Systems and Biotechnology, Peter the Great Saint-Petersburg Polytechnic University, Saint-Petersburg 195251, Russia; 4 Saint-Petersburg State University, Saint-Petersburg 199034, Russia; 5 Hospital Pediatry, Saint-Petersburg State Pediatric Medical University, Saint-Petersburg 194100, Russia; lubov.s.sorokina@gmail.com (L.S.S.); kost-mikhail@yandex.ru (M.M.K.)

**Keywords:** juvenile idiopathic arthritis, cytokines, biomarkers

## Abstract

Juvenile idiopathic arthritis (JIA) is a systemic autoimmune disease that affects the joints, leading to disability. Cytokines and signaling molecules expressed by the immune system cells play a key role in JIA pathogenesis. Understanding how their content changes during pathology development can open up new opportunities for its diagnosis and treatment. The blood plasma of 30 patients with JIA (14 males and 16 females with a mean age of 12.2 ± 4.1) and 20 relatively healthy individuals (10 males and 10 females with a mean age of 10.20 ± 5.85) was analyzed to determine the levels of cytokines using the MILLIPLEX^®^ kit. An increase in interleukins (IL)-1α, 1β, 2, 4, 5, 6, 7, 8, 9, 10, 13, 15, 17F, 22, and 27 and a decrease in IL-3 levels have been shown in patients with JIA. Levels of cytokines, which are important for B-cell activation and proliferation, are increased, while levels of T-cell activating factors remained similar to the control group. Based on our results, it can be assumed that the use of combination therapy aimed at inhibiting both nonspecific interleukins and cytokines that activate B-cells will be more effective for the treatment of JIA.

## 1. Introduction

Juvenile idiopathic arthritis (JIA) is a polygenic systemic autoimmune disease with a genetic component. It is characterized by inflammatory processes in the joints and in vital organs such as the heart, kidneys, and liver. JIA affects children under 16 years old. JIA strongly affects the patient’s quality of life since chronic inflammation limits the patient’s daily activities and productivity [1]. It also can lead to partial or complete disability, as well as a shorter life expectancy. The prevalence of JIA reported by studies varies widely due to a lack of uniform classification methods. Moreover, there are large variations in the frequency of the disease reported by region. Some sources report that the incidence ranges from 1 to 22 and that the prevalence of the disease ranges from 7 to 150 cases per 100,000 children [2]. A study conducted in Turkey showed that the prevalence of JIA during childhood was 64 cases per 100,000 children [3], whereas in Australia, there were up to 400 cases of JIA per 100,000 children [4]. However, such difficulty in treating JIA is probably due to the lack of examination and the lack of high-quality, timely, and comprehensive diagnosis of this autoimmune disease.

Despite the diversity of body systems and the complexity of pathogens involved, autoimmune diseases often have similar clinical symptoms and a common etiology. These conditions are also characterized by symptomatic mimicry with diseases of a different nature. The similarity of symptoms complicates early diagnosis and timely administration of effective treatment.

Since it is impossible to visually distinguish autoimmune diseases from other pathologies with similar symptoms, laboratory diagnostics are often used to determine the occurrence of autoimmune diseases.

Laboratory diagnostics include markers such as erythrocyte sedimentation rate (ESR)—a marker of non-specific inflammation [5], antinuclear antibodies (ANA), anti-citrullinated peptide antibodies (ACPA), and rheumatoid factor (RF). ANA is not an effective marker because of its false positivity and transient positivity (e.g., secondary to infections) [6], and ACPA and RF are usually absent in patients with JIA [7].

Thus, current diagnostics do not allow us to accurately measure the development of JIA. However, the determination of the levels of various interleukins (ILs) could be helpful in finding specific markers.

Many autoimmune diseases are currently being treated with antibodies that inhibit the actions of certain cytokines, such as (IL-6) or tumor necrosis factor (TNF). However, during the development of a child’s body, there is an active proliferation of cells, which requires enhanced control by the immune system. To avoid the occurrence of immunodeficiency, the use of these drugs is strictly limited; therefore, the study of a wide range of IL content can provide promising new methods for the treatment of JIA.

ILs are a group of cytokines that are expressed and act primarily on leukocytes, but some of them also act on other cells. For example, there is a group of cytokines, including IL-1, IL-6, and TNF-α, for which the functions have been fully described by many researchers, especially regarding their role in rheumatic diseases [8,9,10,11,12]. In this paper, other representatives of interleukins are described.

Although many studies have been conducted on common biochemical markers of JIA, studies on individual markers are still lacking.

IL-2 is one of the most important cytokines secreted by T-cells and has the following effects on the cells of the immune system [13]:

1. Causes the expansion of antigen-specific clones (T-helpers which have clusters of differentiation-4 (CD4), T-killers (CD8)).
2. Increases production of other cytokines (T-helpers (CD4), T-killers (CD8), and natural killer (NK) cells).
3. Required for differentiation in the T-helper1 and T-helper2 (Th1 and Th2) subsets, as well as for the development and differentiation of T regulatory cells (T-helpers (CD4)).
4. Increases cytolytic activity (T-killers (CD8) and NK cells).
5. Promotes proliferation (B-cells and NK cells).
6. Enhances the secretion of antibodies and initiates transcription of immunoglobulin J-chain in B-cells.
7. Induces proliferation of memory cells (T-killers (CD8)) [14].

IL-3 is a cytokine that regulates hematopoiesis, but it also has other functions—increasing antigen for T-cell responses, increasing cytotoxicity and adhesion of macrophages, promoting the function of eosinophils, basophils, and mast cells, and playing a critical role in some forms of delayed-type hypersensitivity [15].

IL-4 is primarily responsible for the stimulation of Th0 to Th2 differentiation, but it also acts on B-cells, initiating their growth, differentiation, and immunoglobulin E (IgE) isotype switching. It also activates the expression of major histocompatibility complex class 2 on B-cells. In rheumatic diseases, it has been shown to inhibit the secretion of pro-inflammatory molecules, and prostaglandins, as well as the proliferation of synoviocytes [16].

IL-5 was initially characterized as an inducer of B-cell differentiation into plasma cells [17]. This IL is produced by both hematopoietic and non-hematopoietic cells, including B-cells, basophils, and eosinophils and they are also its targets. IL-5 maintains survival and stimulates the differentiation of B-cells and eosinophils. Therefore, it is considered to be a promising marker and therapeutic target, for example, in bronchial asthma [18].

IL-7 plays an important role in the development and reproduction of all lymphoid cell types [19], stimulates T-cells, B-cells, and the common lymphoid progenitor differentiation [20]. Long-lived memory T cells are differentiated under the influence of IL-7.

IL-8 is a chemokine of neutrophils, causing their migration into tissues [21], as well as their activation. It is expressed by macrophages, monocytes, endotheliocytes, keratinocytes, and dendritic cells. IL-8 is widely studied in oncology since IL-8 keeps dendritic cells near tumors, preventing them from migrating to the lymph node [22].

IL-9 stimulates the proliferation of immune cells, inhibits apoptosis of immune cells, and causes the growth of osteoclasts in rheumatoid arthritis [23]. Recently, some researchers found that this cytokine also acts on T regulatory cells and affects the differentiation of Th1/Th17 [24].

IL-10 is the main anti-inflammatory cytokine. Since the main task of the immune system is to destroy the pathogen and minimize damage to its own tissues, IL-10 is produced by macrophages, monocytes, dendritic cells, neutrophils, mast cells, eosinophils, and natural killers, as well as CD4+, CD8+ T-, and B-lymphocytes [25]. Tumor cells often produce this cytokine, which allows them to camouflage themselves from immune cells. Crohn’s disease is characterized by a deficiency of IL-10 [26].

IL-12 is produced by dendritic cells, macrophages, and B-cells [27]. It induces the production of interferon-γ (IFNγ) and T helper cell differentiation into the Th1 population [28].

IL-13 plays an important role in the development of allergic reactions, but it also reduces the production of receptor activator of the nuclear factor kappa-B ligand (RANKL), thereby reducing bone resorption, inactivating the matrix metalloprotease (MMP) 2 and MMP9, thus preventing cartilage destruction, and inhibiting angiogenesis. It also reduces the production of pro-inflammatory cytokines [29]. Macrophages differentiate into the M2 phenotype after stimulation by IL-13.

IL-15 belongs to the IL-2 family; therefore, it has properties similar to IL-2. However, IL-15 differs substantially from other IL-2 cytokines because it affects the survival of T-killers, natural killers, and neutrophils, and stimulates the proliferation of B-cells and their homing [30]. Recent research has shown that it may be an early link in the inflammatory cascades of various pro-inflammatory cytokines [31].

The role of IL-17 in the development of some autoimmune pathologies has already been studied. It is mainly secreted by Th17 cells, autocrine stimulating the differentiation of these cells, but, under certain conditions, it can also be secreted by other immune cells [32]. IL-17 induces the production of pro-inflammatory cytokines and chemokines by various cells, recruits neutrophils, and activates lymphocytes and macrophages [33]. Normally, the role of IL-17 is to stimulate the antibacterial immune response, while in rheumatic diseases, it promotes the positive feedback loop of IL-6 expression, which is associated with its ability to induce systemic inflammation, characteristic of the development of many autoimmune pathologies [34].

IL-18 (IFNγ inducing factor) was discovered in 1989 as a previously unknown pleiotropic factor. It induces the production of IFNγ, TNF-α, IL-1, IL-2, adhesion molecules, and apoptosis factors, increases the proliferative activity of T-lymphocytes, the activity of natural killer cells (NK), and promotes the differentiation of monocytes/macrophages into osteoclasts, which leads to joint destruction and bone resorption [35].

IL-22 is synthesized by lymphoid cells and has both pro-inflammatory and anti-inflammatory effects [36]. Under physiological conditions, it plays a key role in wound healing, induces angiogenesis, and stimulates cholesterol metabolism [37]. In microbial infections, it stimulates survival, proliferation, and production of innate antimicrobials by epithelial cells, while chronic overexpression in healthy tissues can lead to hyperproliferation, and production of chemokines and other inflammatory signals, ensuring subsequent recruitment of pathological effector cells into inflamed tissues [38]. It also influences Th0, stimulating their differentiation into Th17 [39].

IL-27 is secreted by monocytes, macrophages, lymphocytes, activated dendritic cells, plasma cells, natural killer cells, and other non-immune cells such as placental trophoblasts and endothelial cells. It is involved in the differentiation of Th0 cells into Th1, inhibiting the growth of other subpopulations [40] and the development of osteoclasts and Th17, which has been found in samples from patients with rheumatoid arthritis [41].

These cytokines are involved in pathological and physiological processes. For example, TNF-α causes the death of cells that are affected by viruses; IL-1 and IL-6 are the main factors in physiological immune responses and are thus present in healthy patients. In this regard, it is important to analyze a wide range of cytokines in JIA patients, which can lead to the development of more specific diagnostic markers and targets for treatment.

The purpose of our study was to evaluate a wide range of cytokines in JIA and to select those that would demonstrate what type of immune response is predominant in this disease.

## 2. Materials and Methods

Heparinized blood plasma of 30 patients with a diagnosis of JIA (JIA) and 20 conditionally healthy patients (control group, CG) was used. The main selection criterion for the control group was the absence of autoimmune and infectious diseases. There were 14 male and 16 female patients in the JIA group and 10 male and 10 female patients in the control group. The mean age of patients in the JIA group was 12.2 ± 4.1 years and 10.2 ± 5.9 years for patients in the control group. All JIA patients received non-specific treatment: methotrexate or corticosteroids.

Enzyme-linked immunosorbent assay was used to determine the levels of cytokines, namely the kit MILLIPLEX^®^ Human Cytokine/Chemokine/Growth Factor Panel A Magnetic Bead Panel (MILLIPLEX, Merck KGaA, Darmstadt, Germany).

All analyzed ILs were conventionally divided into 3 groups:

1. Common cytokines, including ILs, the change in the levels of which in patients diagnosed with JIA was previously described in the literature and currently serves to confirm the diagnosis: IL-1α, 1ß, 6, and 18 and TNF-α.
2. Proinflammatory cytokines: IL-2, 3, 5, 7, 8, 9, 12(p40), 15, 17, and 22, which are divided into the following subgroups:
  - IL-2 family: IL-2, 7, 9, and 15
  - IL-3 family: IL-3 and IL-5
  - Not integrated into families: IL-8, 12(p40), 17, and 22
3. Anti-inflammatory cytokines: IL-4, 10, 13, and 27

Statistical analysis: The sample size was not calculated initially. Statistical processing for the comparison of quantitative characteristics for the two groups was carried out using the GraphPad Prism 8 (GraphPad Software, Inc., Boston, MA, USA) software package. The distribution normality was determined by the Shapiro–Wilk test. Differences between groups were determined by Welch’s *t*-test with a normal distribution, and the Mann–Whitney test for a non-normal distribution, with a significance level of less than 0.05 (*p* < 0.05). The figures were generated in GraphPad Prism 8.

## 3. Results

### 3.1. Content of the Common Cytokines

**Figure 1:**
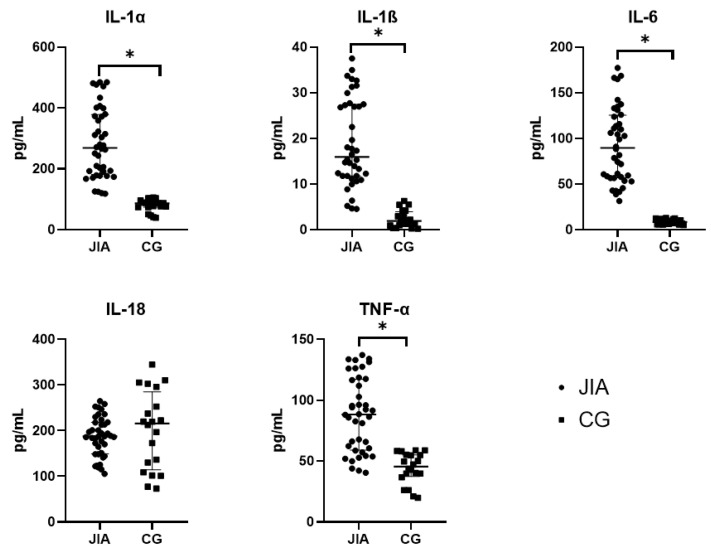
Levels of IL-1α, 1ß, 6, and 18 and TNF-α in the blood plasma of patients with JIA and the control group. Abbreviations: JIA—juvenile idiopathic arthritis, CG—control group, IL—interleukin, and TNF-α—tumor necrosis factor-α. The data are presented as Me (Q1 and Q3). Me is graphically indicated by horizontal lines. Whisker plots indicate interquartile distances. * Differences between groups in the cytokines presented are significant according to the Mann–Whitney test (*p* < 0.05).

In this research, the levels of IL-1α, 1ß, and 6 and TNF-α was found to be significantly higher in the group of patients diagnosed with JIA compared to the group of conditionally healthy patients (Table 1). This is consistent with the results of other studies and allows us to validate the experiment which was conducted using MILLIPLEX ELISA (MILLIPLEX, Merck KGaA, Darmstadt, Germany). All presented values have statistically significant differences with a significance level less than 0.05 (*p* < 0.05) according to the Mann–Whitney test. The level of IL-18 in the blood plasma of JIA patients is similar to that of the control group, which is also comparable with data in the literature, indicating that it is significantly increased in diseases with similar symptoms—Still’s syndrome. Meanwhile, such changes were not observed previously for destructive bone injuries in rheumatoid arthritis and JIA patients. Figure 1 presents the data obtained for the cytokines described above.

### 3.2. Content of the Proinflammatory Cytokines

The assessment of the cytokine profile provides information about the levels of a large number of proinflammatory ILs and makes it possible to separate JIA from other pathologies. It also helps to understand the fundamental mechanisms of its development. The analysis of the content of this cytokine group, which is divided into subgroups, is discussed below.

#### 3.2.1. Content of the IL-2 Family

This family includes IL-2, IL-4, IL-7, IL-9, and IL-15, which mediate effects through the IL-2α (CD25), IL-2β (CD122), and γc (common gamma chain) receptors. IL-4, based on its anti-inflammatory properties and functions, is described in detail in the section about anti-inflammatory cytokines.

**Figure 2:**
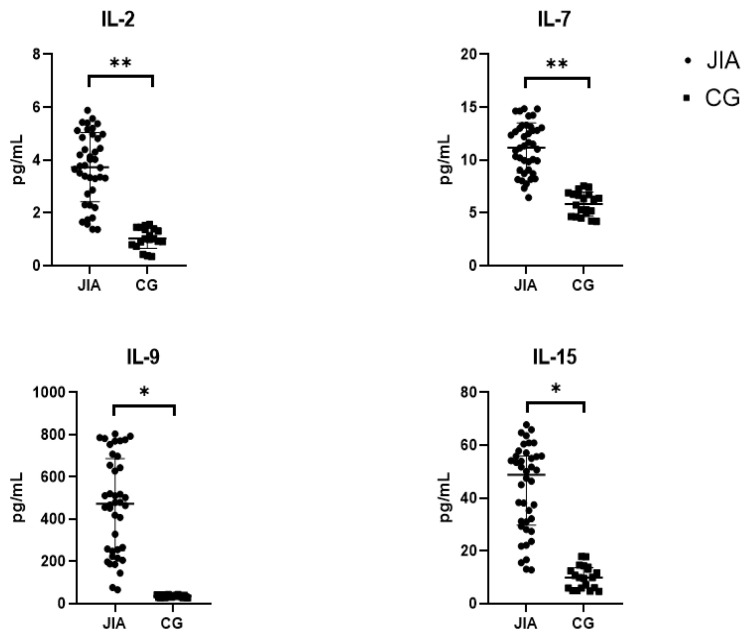
Levels of IL-2 cytokines in the blood plasma of patients with JIA and the control group. Abbreviations: JIA—juvenile idiopathic arthritis, CG—control group, and IL—interleukin. The data are presented as Me (Q1 and Q3) for IL-9 and IL-15. Mean values ± SD are listed for IL-2 and IL-7. Me is graphically indicated by horizontal lines. Whisker plots indicate interquartile distances. * Differences between groups in the cytokines presented are significant according to the Mann–Whitney test (*p* < 0.05). ** Differences between groups in the cytokines presented are significant according to the Welch’s *t*-test (*p* < 0.05).

The results of this study showed a statistically significant (*p* < 0.05) increase in the content of all IL-2 family members in JIA compared to CG. Welch’s *t*-test was used to analyze IL-2 and IL-7, and the Mann–Whitney test was used to analyze IL-9 and IL-15 due to a non-normal distribution of the data. The results of this study are presented in Table 2 as Mean ± SD for IL-2, IL-7, and IL-15 and Me (Q1 and Q3) for IL-9, also shown in Figure 2.

#### 3.2.2. IL-3 Family

**Figure 3:**
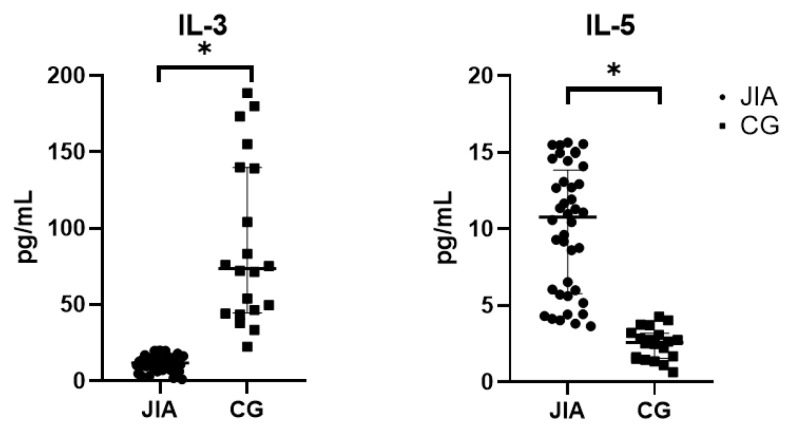
Levels of IL-3 and IL-5 cytokines in the blood plasma of patients of the studied groups. Abbreviations: JIA—juvenile idiopathic arthritis, CG—control group, and IL—interleukin. The data are presented as Me (Q1 and Q3). Me is graphically indicated by horizontal lines. Whisker plots indicate interquartile distances. * Differences between groups in the cytokines presented are significant according to the Mann–Whitney test (*p* < 0.05).

This group includes IL-3 and IL-5, which regulate hematopoiesis. IL-5 is directed predominantly to the eosinophil lineage and B-cells, inducing antibody secretion. IL-3 is secreted due to hypersensitivity reactions and acts on mast cells, basophils, and platelets, while IL-5 acts systemically, attracting eosinophils. Our study showed that the level of IL-3 in the blood plasma of JIA patients is significantly lower than that of the CG patients (*p* < 0.05), whereas IL-5 is significantly higher in JIA patients compared to the control group (*p* < 0.05). Data are presented in Table 3 and Figure 3.

IL-5 is secreted in large quantities by Th2, which can induce type 2 inflammation and tissue damage. An increase in IL-5 in JIA patients can serve as a marker indicating the autoimmune nature of inflammatory bone damage in the case of arthritis of an unspecified nature. This can potentially increase the speed and accuracy of diagnosis and lead to optimal treatment.

#### 3.2.3. Cytokines Which Are Not Integrated into Families 

**Figure 4:**
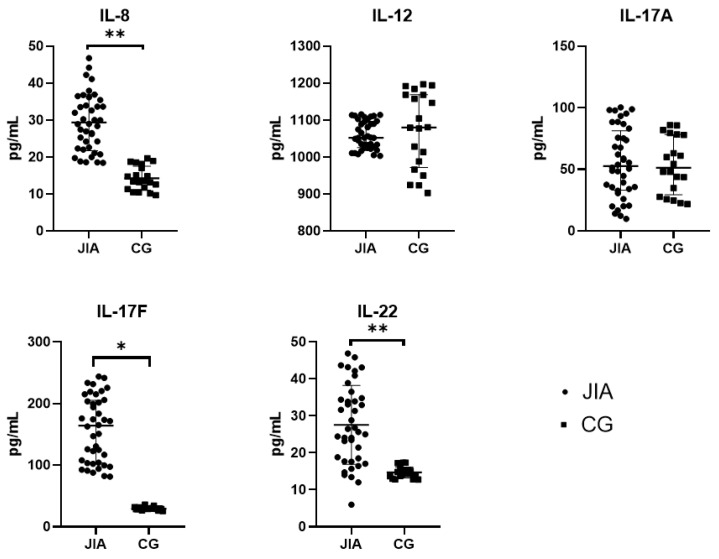
Levels of IL-8, IL-12(p40), IL-17A, IL-17F, and IL-22 in the blood plasma of JIA patients and the control group. Abbreviations: JIA—juvenile idiopathic arthritis, CG—control group, and IL—interleukin. The data are presented as Me (Q1 and Q3) for IL-17F and IL-12(p40). Mean values ± SD are listed for IL-8, IL-17A and IL-22. Me is graphically indicated by horizontal lines. Whisker plots indicate interquartile distances. * Differences between groups in the cytokines presented are significant according to the Mann–Whitney test (*p* < 0.05). ** Differences between groups in the cytokines presented are significant according to the Welch’s *t*-test (*p* < 0.05).

This group includes IL-8, IL-12(p40), IL-17, and IL-22. IL-17 is the common name for two cytokines similar in structure and function: IL-17A and IL-17F. The difference between the two cytokines is that IL-17F is expressed by a large number of different cells. According to the results of this study, the levels of IL-8, IL-17F, and IL-22 were significantly higher in patients with JIA compared with CG according to the Welch’s *t*-test (*p* < 0.05), while the levels of IL-12(p40) and IL-17A were similar to CG (Table 4 and Figure 4).

The difference in the results for IL-17A and IL-17F can be used as a basis for distinguishing JIA from psoriatic arthritis, in which both blood cytokine levels are elevated.

### 3.3. Anti-Inflammatory Cytokines

**Figure 5:**
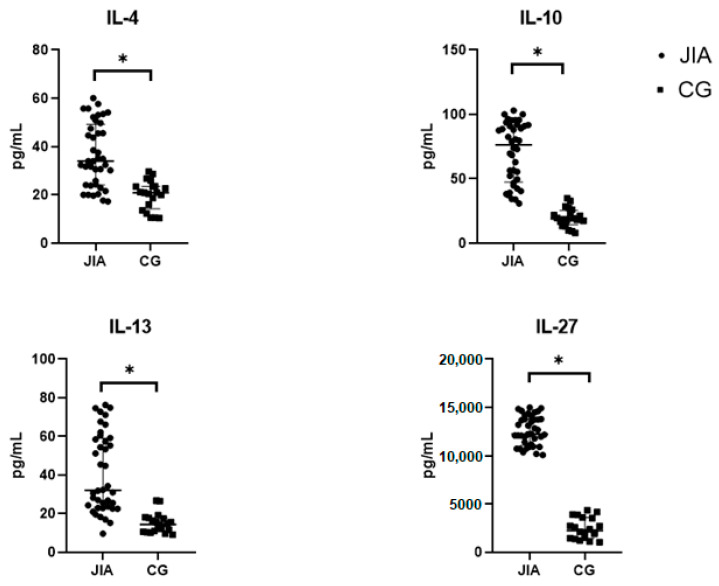
Levels of IL-4, IL-10, IL-13, and IL-27 in the blood plasma of JIA and the control group. Abbreviations: JIA—juvenile idiopathic arthritis, CG—control group, and IL—interleukin. The data are presented as Me (Q1 and Q3). Me is graphically indicated by horizontal lines. Whisker plots indicate interquartile distances. * Differences between groups in the cytokines presented are significant according to the Mann–Whitney test (*p* < 0.05).

For a full assessment of the inflammatory process, it was necessary to analyze the levels of pro-inflammatory as well as anti-inflammatory cytokines such as IL-4, IL-10, IL-13, and IL-27. This division is arbitrary because the final effect of these molecules depends on the environment and the content of other signaling compounds. In JIA patients, a significant increase was observed in the levels of IL-4 and IL-27 according to Welch’s *t*-test (*p* < 0.05), and in the levels of IL-10 and IL-13 according to the Mann–Whitney test (*p* < 0.05) (Table 5 and Figure 5).

Our results demonstrated large differences in the IL-27 content between JIA and CG patients, which should be taken into account in future comprehensive studies, both for diagnosis and for assessing the activity of the disease. The content of this cytokine can be very informative for the development of a multicomponent diagnostic system.

## 4. Discussion

JIA is a serious autoimmune disease with multifactorial etiology and has not been well-studied; it is characterized by arthralgia lasting at least two weeks (in the absence of arthritis) or in most cases, with arthritis. In the presence of JIA, as in other autoimmune pathologies, a normally functioning immune system typically consisting of dynamic components that allow quick and accurate responses to external (pathogens) and internal (for example, cancer cells) threats enters an imbalance and begins to attack its own organs and tissues. Such changes are mainly due to the dysregulation of immune mediators such as cytokines and chemokines, which leads to a loss of homeostasis and triggers inflammation in an autoimmune manner [42]. In the literature, the participation of cytokines such as TNF-α, IL-1β, and IL-6 in the pathogenesis of JIA is widely discussed [8,9,10,12,43,44], and their studies have evaluated the role of new cytokines in this process, such as IL-35 and IL-36 [45].

Few studies have examined JIA cytokine profiles [46,47,48]. Some of the cytokines mentioned in these papers are similar to those examined in this study. However, it is also important to note that these papers were based on meta-analyses of studies on individual cytokine segments which were conducted by different groups of researchers, using different methods and groups of patients. Unlike our study, they did not conduct a comprehensive analysis of a wide range of cytokines using one method across all study groups, which was a limitation in the other studies and prevented them from determining the importance of the changes in different cytokines.

In order to fully assess the presence of interrelated changes, it is necessary to conduct studies of a wide range of cytokines in a single study. In particular, a similar attempt was made in a study that examined chronic non-bacterial osteomyelitis [49]. Physiological imbalance in pro- and anti-inflammatory cytokines is actively considered by scientists mainly for the development of new treatment methods. We aimed to investigate a wide range of cytokines in order to form a list highlighting key information for all of them, which can provide insights into which mechanisms contribute most to the development of JIA.

Our results showed that in the presence of JIA, there is an increase in the levels of cytokines such as IL-2, IL-4, IL-5, and IL-13, which mediate and regulate adaptive humoral immune responses [15,17,18,19,30]. The levels of most cytokines responsible for the activation of killer cells is reduced or is similar to that of the control group. These data may be useful for the development of more specific therapy aimed at inhibiting cytokines that activate this type of immune response. Although IL-4 inhibits the metalloproteinases synthesis and stimulates metalloproteinases-1 tissue inhibitor production, leading to cartilage destruction prevention [50], an increased level of this cytokine, noted in our study, should probably be regarded as a compensatory reaction to ongoing inflammation in junctions.

Moreover, this study describes several ILs, which levels were at least three times higher (IL-5, 9, 10, 15, 17F, and 27) in the presence of JIA. In the future, the determination of the content of these cytokines may be used to improve the existing recommendations for JIA diagnostics.

The cytokines presented in the work were not chosen randomly, but due to the fact that their function and role in the immune reactions are well-studied and because some of the selected cytokines are targets for anticytokine therapy.

## 5. Conclusions

Our data indicate the role of cytokine imbalance in the pathogenesis of JIA. The increased level of some pro- and anti-inflammatory cytokines confirms the role of immunity humoral link in JIA patients.

It is important to conduct a broad screening of the cytokine profile in JIA in order to understand better the mechanisms underlying the pathogenesis of this disease. For the first time, elevated levels of several cytokines have been demonstrated to have a key role in humoral immunity in the pathogenesis of JIA.

## Figures and Tables

**Table 1:** Levels of IL-1α, 1ß, 6, and 18 and TNF-α in the blood plasma of JIA patients.

	IL-1α (pg/mL)	IL-1ß (pg/mL)	IL-6 (pg/mL)	IL-18 (pg/mL)	TNF-α (pg/mL)
JIA	278.4 (188.1; 375.0) *	17.31 (11.79; 28.82) *	102.8 (56.58; 132.1) *	185.1 (148.5; 205.1)	88.29 (60.05; 126.2) *
CG	87.0 (73.7; 93.7)	1.93 (0.95; 3.9)	8.37 (6.0; 11.7)	216.0 (114.2; 285.1)	45.4 (37.5; 55.1)

Abbreviations: JIA—juvenile idiopathic arthritis, CG—control group, IL—interleukin, and TNF-α—tumor necrosis factor-α. The data are presented as Me (Q1 and Q3). * Differences between groups in the cytokines presented are significant according to the Mann–Whitney test (*p* < 0.05).

**Table 2:** Levels of IL-2 cytokines in the blood plasma of JIA patients.

	IL-2 (pg/mL)	IL-7 (pg/mL)	IL-9 (pg/mL)	IL-15 (pg/mL)
JIA	3.7 ± 1.3 **	11.1 ± 2.3 **	472.9 (242.2; 719.5) *	43.0 ± 15.4 **
CG	1.0 ± 0.4	5.8 ± 1.1	35.7 (29.8; 40.2)	10.0 ± 4.4

Abbreviations: JIA—juvenile idiopathic arthritis, CG—control group, and IL—interleukin. The data are presented as Me (Q1 and Q3) for IL-9 and as Mean ± SD for IL-2, IL-7, and IL-15. * Differences between groups in the cytokines presented are significant according to the Mann–Whitney test (*p* < 0.05). ** Differences between groups in the cytokines presented are significant according to the Welch’s *t*-test (*p* < 0.05).

**Table 3:** Levels of IL-3 cytokines in the blood plasma of JIA patients.

	IL-3, pg/mL	IL-5, pg/mL
JIA	11.8 (7.0; 16.4) *	11.0 (5.5; 13.4) *
CG	73.6 (44.7; 139.8)	2.6 (1.6; 3.2)

Abbreviations: JIA—juvenile idiopathic arthritis, CG—control group, and IL—interleukin. The data are presented as Me (Q1 and Q3). * Differences between groups in the cytokines presented are significant according to the Mann–Whitney test (*p* < 0.05).

**Table 4:** Levels of IL-8, IL-12(p40), IL-17A, IL-17F, and IL-22 in the blood plasma of JIA patients.

	IL-8 (pg/mL)	IL-12(p40) (pg/mL)	IL-17A (pg/mL)	IL-17F (pg/mL)	IL-22, (pg/mL)
JIA	29.0 ± 8.1 **	1075 (1026; 1102)	53.1 ± 29.1	158.0 (106.8; 216.1) *	27.8 ± 11.4 **
CG	14.3 ± 3.3	1080 (971; 1169)	53.4 ± 22.7	29.3 (28.7; 30.7)	14.7 ± 1.5

Abbreviations: JIA—juvenile idiopathic arthritis, CG—control group, and IL—interleukin. The data are presented as Me (Q1 and Q3) for IL-12(p40) and IL-17F and as Mean ± SD for IL-8, IL-17A, and IL-22. * Differences between groups in the cytokines presented are significant according to the Mann–Whitney test (*p* < 0.05). ** Differences between groups in the cytokines presented are significant according to the Welch’s *t*-test (*p* < 0.05).

**Table 5:** Levels of IL-4, IL-10, IL-13, and IL-27 in the blood plasma of JIA patients.

	IL-4 (pg/mL)	IL-10 (pg/mL)	IL-13 (pg/mL)	IL-27 (pg/mL)
JIA	34.0 ± 12.4 **	73.5 (48.9; 91.2) *	31.2 (22.9; 59.0) *	12,215 (11,130; 13,859) *
CG	20.0 ± 6.0	19.0 (14.2; 25.3)	14.5 (10.7; 17.7)	2240 (1467; 3595)

Abbreviations: JIA—juvenile idiopathic arthritis, CG—control group, and IL—interleukin. The data are presented as Me (Q1 and Q3) and as Mean ± SD for IL-4. * Differences between groups in the cytokines presented are significant according to the Mann–Whitney test (*p* < 0.05). ** Differences between groups in the cytokines presented are significant according to the Welch’s *t*-test (*p* < 0.05).

## Data Availability

The raw data supporting the conclusions of this article will be made available by the authors on request.
